# Age-Related Differences in Corticospinal Excitability and Anticipatory Postural Adjustments of the Trunk

**DOI:** 10.3389/fnagi.2021.718784

**Published:** 2021-08-17

**Authors:** Rebecca S. Rowland, Ned Jenkinson, Shin-Yi Chiou

**Affiliations:** ^1^School of Sport, Exercise, and Rehabilitation Sciences, University of Birmingham, Birmingham, United Kingdom; ^2^Centre for Human Brain Health, University of Birmingham, Birmingham, United Kingdom; ^3^Medical Research Council ‘Versus’ Arthritis Centre for Musculoskeletal Ageing Research, University of Birmingham, Birmingham, United Kingdom

**Keywords:** corticospinal excitability, motor evoked potentials, erector spinae, anticipatory postural adjustments, balance, older adults, electromyography

## Abstract

Anticipatory postural adjustments (APAs) are a feedforward mechanism for the maintenance of postural stability and are delayed in old adults. We previously showed in young adults that APAs of the trunk induced by a fast shoulder movement were mediated, at least in part, by a cortical mechanism. However, it remains unclear the relationship between delayed APAs and motor cortical excitability in ageing. Using transcranial magnetic stimulation we examined motor evoked potentials (MEPs) of the erector spinae (ES) muscles in healthy young and old adults prior to a fast shoulder flexion task. A recognition reaction time (RRT) paradigm was used where participants responded to a visual stimulus by flexing their shoulders bilaterally as fast as possible. The activity of bilateral anterior deltoid (AD) and ES muscles was recorded using electromyography (EMG). The onset of AD and ES EMG was measured to represent RRT and APAs, respectively. We found increases in amplitudes of ES MEPs at 40 ms than 50 ms prior to the EMG onset of the AD in both groups. The amplitude of ES MEPs at 40 ms prior to the onset of AD EMG correlated with the onset of ES activity counterbalancing the perturbation induced by the shoulder task in the elderly participants only. Our findings suggest that timing of increasing corticospinal excitability prior to a self-paced perturbation becomes more relevant with ageing in modulating postural control of the trunk.

## Introduction

Postural control involves the ability to maintain one’s center of mass (COM) over the base of support in response to perturbation (Hess et al., 2006). There are two main processes used to restore or maintain postural stability, anticipatory postural adjustments (APAs), and compensatory postural adjustments (CPAs). APAs occur prior to movements and are a feedforward process that counteract an expected perturbation (Alexandrov et al., 2005; Hall et al., 2010). It is considered as a first line of defence for postural stability in anticipation of perturbations and has been observed whilst sitting, standing, or walking (Aruin and Shiratori, 2003). On the contrary, CPAs are responses to external postural perturbation, and therefore under the control of feedback mechanisms (Alexandrov et al., 2005). In contrast to the absence of APAs that can be seen in neurological conditions such as Parkinson’s disease (Bazalgette et al., 1987), it is thought that the APAs in elderly people are delayed, resulting in increased responses of, and reliance upon, CPAs to maintain balance (Kanekar and Aruin, 2014). Examples of delayed APAs are seen in self-paced voluntary shoulder movement (Rogers et al., 1992; Woollacott and Manchester, 1993) where the activity of trunk and lower-limb muscles occurs later than that in anterior deltoid (AD) in over 60 s, whereas the opposite temporal relationship is seen in young adults. While it is recognized that APAs of the trunk in response to a perturbation, measured by electromyography (EMG), are delayed in older adults, it remains largely unclear whether age-related changes in the neural control of APAs exist.

Research has shown that corticospinal excitability of erector spinae (ES) muscles increases prior to a rapid shoulder flexion task (Chiou et al., 2018; Masse-Alarie et al., 2018) and the increased excitability was accompanied by reduced inhibition in M1 with no change in spinal excitability (Chiou et al., 2018). Furthermore, prior work has shown that corticospinal excitability of the ES muscle was increased to a greater extent during an APA eliciting dynamic shoulder flexion task compared with a static shoulder flexion task (with no APAs required) and a goal-directed voluntary trunk extension task in healthy adults (Chiou et al., 2016). The findings provide evidence of motor cortical involvement in modulating APAs of the trunk muscles prior to a self-paced voluntary shoulder movement. The results are in keeping with findings from clinical populations as lesions involving the primary motor cortex (M1), such as stroke (Pereira et al., 2014), are associated with disruptions of APAs in a rapid arm abduction task (Palmer et al., 1996), as well as in a bimanual load-lifting task (Viallet et al., 1992).

Age-related changes in corticospinal excitability in older adults have been reported (Rozand et al., 2019; Skarabot et al., 2019). Using a simple reaction time paradigm a study found that amplitudes of MEPs in a small hand muscle preceding the onset of thumb flexion movements were greater in older adults than in young adults, with no motor slowing in the older adults (Levin et al., 2011). These results indicate age–related changes in corticospinal excitability to a prime-mover prior to movement initiation, albeit with no reported correlations between the corticospinal excitability and reaction time. Furthermore, previous studies reported greater motor cortical activity during postural tasks in older adults (Papegaaij et al., 2014, 2016). For example, motor cortical inhibition in muscles of lower limbs during standing was decreased in older adults compared with young adults and this decreased inhibition correlated with increases in velocity of center of pressure in standing (Papegaaij et al., 2014). While these findings suggest an age-specific relationship between motor cortical activity and postural control, results were obtained during static tasks, such as standing or maintaining a forward lean posture, instead of during a dynamic task. Hence, it remains unknown the extent to which age-related changes in the corticospinal excitability profile of a non-prime mover (i.e., the ES muscle) prior to a dynamic upper limb movement requiring APAs and whether the corticospinal excitability correlates with APAs of the trunk in older adults.

Within this framework, the aims of the study were to compare corticospinal excitability of a trunk muscle (a non-prime mover) prior to a fast, bilateral shoulder flexion movement (i.e., during the APA window) between young and older adults, and to correlate the corticospinal excitability prior to the shoulder movement with APAs of the trunk. We hypothesized that the corticospinal excitability profile during the APA window differs between older and young adults and that the corticospinal excitability correlates with the APAs of the trunk in older adults. To test our hypotheses, we examined amplitudes of MEPs in the ES muscle within the time frame of the APA window prior to the onset of the prime mover (i.e., shoulder flexors), and its relationship with the onset of APAs in healthy old adults and in young adults.

## Materials and Methods

### Ethics Approval

The study was approved by the University of Birmingham Ethics Committee (ERN_17–1541AP4) and performed in accordance with the Declaration of Helsinki. Written informed consent was obtained from all participants prior to the experiment.

### Participants

Twenty-six healthy young adults and 27 healthy older adults (>65 years old) were recruited to the study. The young participants were recruited from the University of Birmingham student body; the elderly participants were recruited through the Birmingham 1,000 Elders Group mailing list. Participants were excluded if they met any of the TMS exclusion criteria (Rossi et al., 2011), including a family history of epilepsy, concussion, pregnancy, metal implants in the brain, unexplained faints, and cardiac pacemakers. Furthermore, participants who did not have visible MEPs in the ES muscle elicited by TMS or were unable to tolerate the sensation of the TMS were also excluded (young: *n* = 6; elderly: = 7). Therefore, there were 20 young and 20 older participants completing the study. All participants had their demographic data taken and completed questionnaires (Table 1). These included the Falls Efficacy Scale—International (FES-I) a 16-item questionnaire measuring individuals concerns or fears of falling (Yardley et al., 2005); the International Physical Questionnaire-Short Form (IPAQ) a 9-item questionnaire measuring the amount of time per week individuals spend on sitting, walking, taking part in moderate, and vigorous activity (Craig et al., 2003); the EuroQol-5D (EQ-5D) a measure of individual’s quality of life, with concerns about their mobility, self-care, pain and discomfort, anxiety and depression, and usual activities, and their self-perceived health based on a visual analogue scale (VAS) between 0 (worst health possible) and 100 (best health possible; Balestroni and Bertolotti, 2012). None of the participants recruited in this study had fallen in the past 12 months. All older participants were able to walk independently without using an aid.

**Table 1:** Demographic data.

	Older participants (*n* = 20)	Young participants (*n* = 20)
Age (years)*	75.45 ± 4.83	20.25 ± 1.41
Height (cm)	169.4 ± 10.94	171.53 ± 7.04
Weight (kg)	64 ± 16.25	68.45 ± 6.86
AMT (%MSO)	81.15 ± 16.99	80.05 ± 14.52
Intensity	97.38 ± 20.39	96.06 ± 17.42
TMS hotpot, with respect to the vertex	Lateral: 2.1 ± 0.8 cm;	Lateral: 2.5 ± 0.9 cm;
Handiness	Right: 17; Left: 3	Right: 19; Left: 1
Hemisphere stimulated	Right: 4; 16 Left	Right: 3; 17 Left
FES-I*
*Fear of falling*	60%	0%
*No fear of falling*	40%	100%
IPAQ*
*METs (min/week)*	2763.3 ± 2568.9	5105.2 ± 5262.7
*Category*		
*High*	25%	75%
*Moderate*	65%	15%
*Low*	10%	10%
EQ-5D
*Mobility*		
*Level 1*	90%	90%
*Level 2*	10%	10%
*Level 3*	0%	0%
*Self-care*	100%	100%
	0%	0%
	0%	0%
*Usual activity*	85%	90%
	15%	10%
	0%	0%
Pain/Discomfort*	45%	85%
	50%	15%
	5%	0%
*Anxiety/Depression*	80%	75%
	20%	25%
	0%	0%
*EQ-VAS (0–100)*	86.4 ± 12.1	84.6 ± 9.3

AMT, active motor threshold; MSO, maximal stimulator output. *P < 0.05, between-group comparisons.

### Electromyography (EMG)

Muscle activity was recorded using surface EMG (Delsys^®^ Bagnoli-2 EMG system). Adhesive electrodes (19.8 mm × 35 mm) were placed over muscle bellies and in line with the approximate orientation of muscle fibers of bilateral ES at the 12th thoracic vertebral level, and AD. Reference electrodes were placed at the 7th cervical vertebral level, and the elbow (olecranon) to reduce background noise. T12 was the chosen point for electrode positioning as ES muscle function in this area is predominantly concerned with postural adjustments to maintain the COM (Davey et al., 2002). EMG signals were pre-amplified (1,000×), and band-passed filtered between 20 and 450 Hz before being sampled at 1 kHz by a micro 1401 ADC (CED, Cambridge, UK). The data were acquired, stored, and analyzed by Signal software (Version 6, CED, Cambridge, UK).

### Experimental Procedures

**Figure 1:**
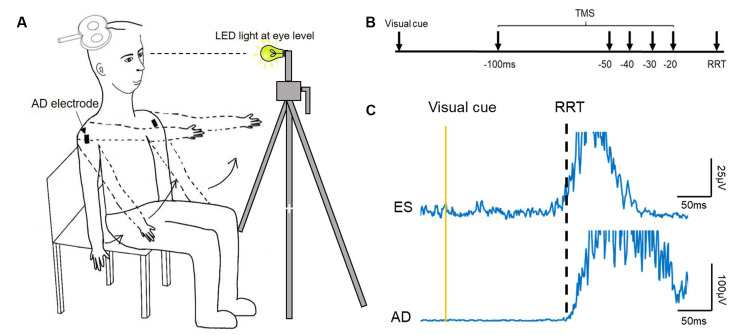
Experimental setup. **(A)** Schematic illustration of the setup of data collection. **(B)** The recognition reaction time (RRT) paradigm and time points where transcranial magnetic simulation (TMS) was given with respect to the expected RRT in the window of anticipatory postural adjustments (APAs). **(C)** Raw electromyography (EMG) traces recorded from left erector spinae (ES) and right anterior deltoid (AD) of a representative young participant during rapid shoulder flexion. Note that the EMG onset of the ES and AD is about the same.

Participants were seated upright in a chair, with arms by the sides of the body and the torso unsupported (Figure 1A). A seated position was chosen due to our preliminary data (*n* = 6) showing no significant differences in the timing of increasing corticospinal excitability of the ES between a seated or standing position in healthy young adults. This finding is in line with previous research showing no differences in corticospinal excitability between different postures, such as between standing or lying (Chiou et al., 2018).

Individual reaction time was determined using a recognition reaction time (RRT) protocol (Ziemann et al., 1997), where false stimuli mix with one correct stimulus promoting the response. It consisted of two conditions: (1) LED light (correct stimulus), and (2) TMS click (false stimulus). Participants were instructed to flex bilateral shoulders to 90° as fast as they could when the LED light flashed and to stay still (arms kept by their side with no movement) during the TMS click condition. The LED light was positioned ~1 m from the participant at eye level and controlled by a button press (Figure 1); the TMS coil was held away from the participant’s head. A verbal warning signal, ‘Ready’ was given to all participants prior to the LED light or TMS click. The interval between the warning signal and the LED light was unfixed, varying between 1–5 s. The RRT protocol was used to ensure that the participant reacted only to the LED light and not to the TMS click, as this type of reaction time was required in the main experiment. Each condition (click; light) was repeated 10 times and their order was randomized. The 10 trials obtained from the light condition were averaged and an RRT was determined visually at which AD EMG rose above 3 SD of the mean background EMG level in a 50 ms window prior to the visual cue (Chiou et al., 2018; Hortobagyi et al., 2018). This data was then used to calibrate the timing of the TMS stimuli for the main experimental condition for each participant. All participants performed three brief maximal voluntary contractions (MVCs, ~2 s) of the ES in a prone position, with pelvis and ankles fixed securely and resistance provided at the scapula. Consistent verbal encouragement was given to all participants during the MVCs.

### Transcranial Magnetic Stimulation (TMS)

TMS was delivered over the M1 using a Magstim 200^2^ stimulator connected to a D70 Alpha figure-of-eight coil (outside coil diameter: 18 cm; The Magstim Company Limited, UK). The coil was held 45° away from the midline with the handle pointing posteriorly. The optimal position (MEP hotspot) for the coil was identified by moving the coil in small increments in the area. The hotspot was defined as the point in which the largest MEP was elicited by TMS in the contralateral ES muscle. The position of the coil was marked on the scalp with a marker pen to allow consistent placement of the coil thereafter. The procedure started with the hemisphere ipsilateral to the dominant arm, determined by the Edinburgh handiness questionnaire (Oldfield, 1971). If clear ES MEP responses were not obtained, the same procedure was repeated on the other hemisphere. The hemisphere which gave the most reliable MEPs in the contralateral ES muscle was chosen as the targeted hemisphere (Chiou et al., 2016).

### Motor Evoked Potentials (MEPs)

Active motor threshold (AMT) of the ES was assessed while participants were seated upright and defined as the minimal stimulus intensity required to evoke visible MEPs in at least 3/6 consecutive trials. Test intensity was then set to 120% of this AMT. If 120% of AMT was above the maximal stimulator output (i.e., >100%MSO), an intensity of 100%MSO was used (young: 5/20; elderly: 7/20). Ten ES MEPs were evoked while participants were in an upright seated position as the baseline of corticospinal excitability of ES. The stimulus paradigm used in the main experiment was similar to that used in a previous study (Chiou et al., 2018). Briefly, the experimental conditions consisted of seven states: light only (no TMS pulses), TMS only (no light), light followed by TMS stimuli at −100, −50, −40, −30, and −20 ms with respect to the individual’s previously determined reaction time (Figure 1B). The seven states were randomized in one cycle and five cycles were repeated in two separate blocks, with 70 trials in total. In situations where participants reacted to the warning signal or the TMS click, they were required to complete additional trials until they reached 10 cycles with only correct responses. Participants were given as much rest as needed during the main experiment to ensure consistent performance and to avoid fatigue.

### Data Analysis

EMG data were first visually inspected to remove trials without a visible MEP, a failure to respond, or incorrect responses i.e., to TMS only states. Frames where ES or AD activity were present before the stimulation were also removed. The onset time of the AD and ES muscles was measured manually frame-by-frame from the rectified EMG traces and defined as a point where EMG activity rose above more than 3 SD of the mean pre-stimulus EMG level (Hodges and Richardson, 1997). Due to RRT varying throughout the trials, data were reorganized based on the timing of the TMS pulse relative to the onset of the ipsilateral AD muscle, hence no MEPs in the AD, to avoid the inhibitory effect following MEPs (i.e., silent period) on EMG activity (Nikolova et al., 2006; Burns et al., 2017). ES MEPs were sorted in 10–20 ms windows (−100ms, 85 ms, 70 ms, 60 ms, 50 ms, 40 ms, 30 ms, and 20 ms) and their amplitudes were averaged within each window. After reorganizing the data based on the actual timing of the TMS pulse, each participant had four or more MEPs being averaged in each time window (young: 5.3 ± 0.9 MEPs, range: 4–10; old: 5.2 ± 1.0 MEPs, range: 4–10). Data obtained at 20 ms prior to the actual RRT were excluded from analysis due to most frames being contaminated by the presence of background ES activity at that time window in both groups. Signal scripts were also used to calculate peak-to-peak amplitude of MEPs in the ES muscle and expressed as a percentage of the baseline MEP amplitude when participants were not expecting a visual cue. Root-mean-square amplitudes of background EMG (rmsEMG) in a 100 ms window prior to the stimulation from unrectified EMG traces were calculated and expressed as a percentage of MVC.

All questionnaires were analyzed. The FES-I was analyzed through a sum of the total score of all 16 answers, giving a result between 16 and 64. The IPAQ was analyzed through the use of a formula equating the MET (metabolic equivalents) minutes of each score of walking (3.3× minutes per week), moderate activity (4× minutes per week), and vigorous activity (8× minutes per week), referring to the amount of oxygen consumed e.g., 1MET = at rest. EQ-5D was analyzed by categorizing answers into “problem” or “no problem”; score 1 was categorized as no problem and score 2 and above was categorized as problem. Their EQ-VAS (VAS) score was taken from the visual analogue scale (0–100; 0 indicating poor health, 100 indicating good health), indicating their self-rated wellness on the day.

### Statistical Analysis

Statistical Program for the Social Sciences (SPSS v.23, IBM Corp., Armonk, NY, USA) was used to perform statistical analysis. Normal distribution was tested by the Shapiro-Wilk test. If data failed the normality test, non-parametric tests were applied; if sphericity assumption failed for repeated ANOVAs, Greenhouse-Geisser corrections were applied. For continuous data (IPAQ-MET, EQ-VAS, RT), Independent *t*-tests or Mann-Whitney *U* tests (for non-parametric tests) were used to examine between-group differences (young vs. elderly cohort). Paired-*t*-tests or Wilcoxon Signed Ranks tests were applied for within-subject comparisons. For discrete data (IPAQ, EQ-5D; FES-I), Chi-square tests were employed for between-group comparisons. To determine changes in ES MEPs along the course of time in the APA window and whether this was different between groups, a mixed-model repeated measure ANOVA was applied to examine any effect of time (−100 ms, 85 ms, 70 ms, 60 ms, 50 ms, 40 ms, 30 ms, TMS alone) and an interaction between group and time on ES MEPs and background EMG. *Post hoc* tests with Bonferroni’s correction were applied where indicated. To characterize the relationship of corticospinal excitability in the APA window, Spearman’s correlation analyses were performed to examine correlations between amplitudes of ES MEPs and EMG onset of the ES muscle as well as the difference in EMG onset between the ES and AD muscles. Significance was set at *p* < 0.05, with Bonferroni correction to adjust for multiple comparisons if needed. Group data are presented as mean ± SD in the text and mean ± SEM in figures.

## Results

### Delayed Anticipatory Postural Adjustments in the Elderly Group

**Figure 2:**
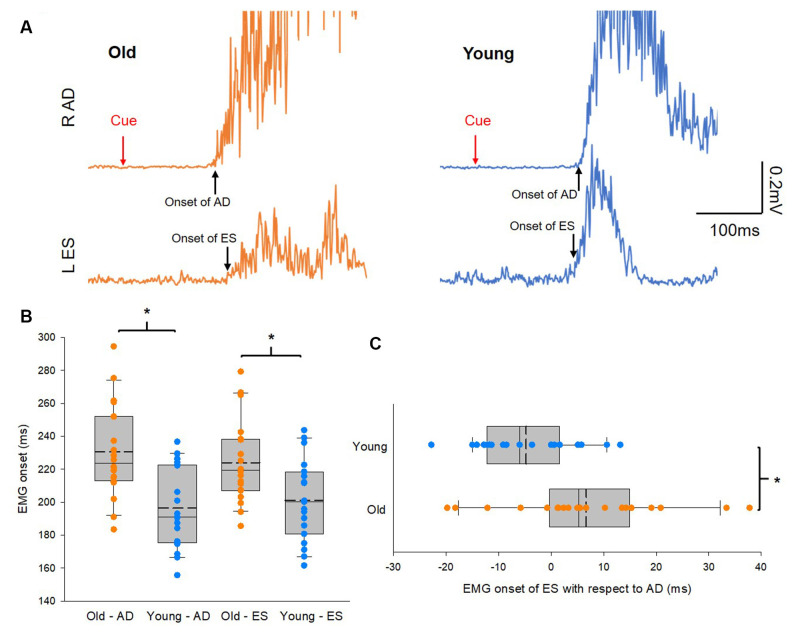
Anticipatory postural adjustments (APAs). **(A)** Electromyography (EMG) traces recorded from left erector spinae (ES) and right anterior deltoid (AD) of representative old and young participants. Traces show the average of 10 EMG traces recorded during rapid shoulder flexion. Note that in the old participant EMG onset of AD preceding to EMG onset of ES, hence delayed APAs. Conversely, EMG onset of ES preceding to EMG onset of AD in the young participant. **(B)** Group data (old: *n* = 20; young: *n* = 20) showing EMG onset of AD and ES. The abscissa shows the muscles and the group and the ordinate shows the onset (ms) of muscle activity calculated from AD and ES with respect to the visual cue. **(C)** Group data (old: *n* = 20; young: *n* = 20) showing EMG onset of ES with respect to EMG onset of AD. The abscissa shows the difference in EMG onset between ES and AD and numbers above 0 mean the onset of ES EMG is later than the onset of AD EMG and* vice versa*. The ordinate shows the group. Solid lines indicate median values; dotted lines indicate mean values. The box is interquartile range; error bars denote maximum and minimum values. *p < 0.05, comparisons between groups.

Figure 2A illustrates traces of averaged 10 EMG activities in AD and ES during the rapid shoulder flexion task from a representative subject of each group. Note that EMG onset in the ES muscle is later than that of the AD muscle in the older participant, while the reverse is true in the young participant, even though the reaction time between the elderly and young participants is similar. Group results showed that onsets of EMG activity in AD and ES were slower in the elderly group (AD: 231 ± 24 ms; ES: 235 ± 30 ms) than in the young group (AD: 205 ± 25 ms; *t*_(38)_ = 3.3, *p* = 0.002; ES: 202 ± 27 ms; *t*_(38)_ = 3.58, *p* = 0.001; Figure 2B). In addition, there was a delay in the onset of EMG activity in the ES with respect to the onset of EMG activity in the AD in the older participants compared with the young participants (Figure 2C). The findings suggest impaired APAs during a rapid shoulder flexion task in our elderly cohort.

### No Difference in Corticospinal Excitability Profile During APAs Between Old and Young Adults

**Figure 3:**
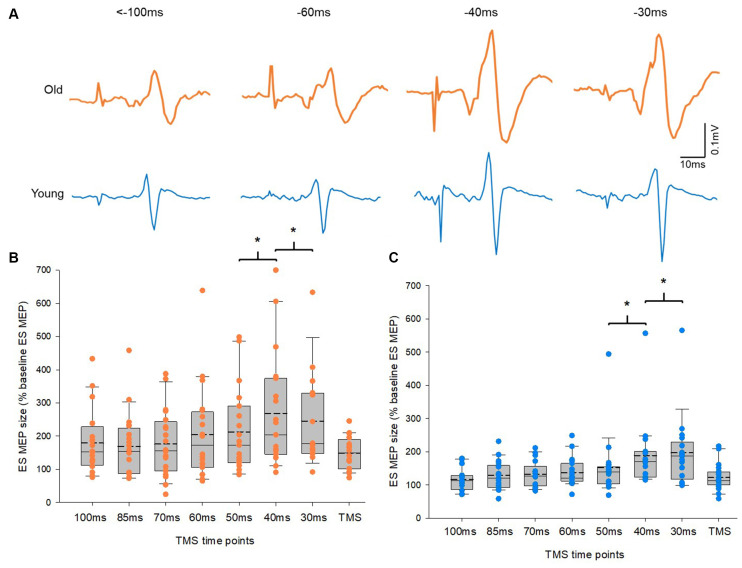
Motor evoked potentials (MEPs) in the erector spinae (ES) muscle. **(A)** MEP traces recorded from the ES muscle of representative old and young participants. Traces show the average of 10 MEPs in the ES muscle at various time points prior to the RRT and within the window of anticipatory postural adjustments (APAs). Group data **(B)** from older participants (*n* = 20) and **(C)** from young participants (*n* = 20) showing changes in MEPs in the ES muscle along the course of time in the APA window. The abscissa shows the time points where transcranial magnetic stimulation (TMS) was given with respect to the actual recognition reaction time (RRT). TMS means trials with no visual cue but pulses of TMS. The ordinate shows the size of ES MEP (as a % of the ES MEP obtained at baseline when participants were not expecting to react to a visual cue). Note that the size of ES MEPs increases significantly at 40 ms and 30 ms prior to the RRT in both old and young participants. The box is interquartile range; error bars denote maximum and minimum values. *p < 0.05, comparisons between time points.

Figure 3A illustrates traces of averaged 10 MEPs in the ES from a representative subject of each group. Note that the size of MEPs in the ES increased at time points closer to the reaction time in both groups. A mixed model repeated measures ANOVA showed a main effect of time on ES MEPs (*F*_2.04,56.98_ = 12.1, *p* < 0.001). However, there was no interaction between time and group in ES MEPs. *Post hoc* tests revealed that the amplitude of ES MEP was greater at −40 ms (data from −49 to −40 ms) than at −50 ms (data from −59 to −50 ms). In addition, the amplitude of ES MEP was greater at −30 ms (data from −39 to −30 ms) than at −40 ms in both groups (Figures 3B,C). There was no difference between other time points from −100ms to −50 ms. The amplitudes of ES MEPs were greater at TMS only than at baseline when participants were not expecting to react to a cue (*Z* = −3.9; *p* < 0.001), suggesting the involvement of attention to the corticospinal excitability. No differences in ES EMG were found between the elderly and young groups across all time points (*F*_3.24,84.35_ = 1.4, *p* = 0.249; elderly: 9.18 ± 3.3% of MVC; young: 5.87 ± 2.60% of MVC). ES MVCs were greater in the young cohort (0.12 ± 0.04 mV) than in the elderly cohort (0.06 ± 0.03 mV; *t*_(32.66)_ = −5.197, *p* < 0.001) as expected.

### EMG Onset of the ES Muscle Correlates With the Size of ES MEP in Older Adults

**Figure 4:**
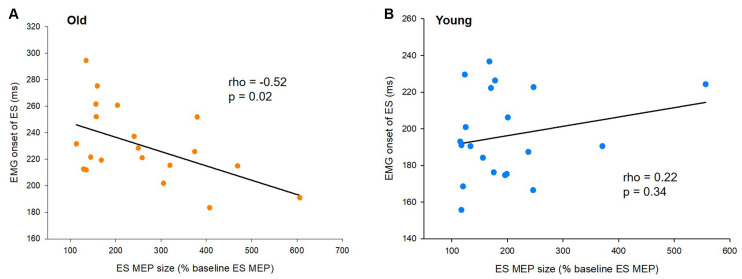
Motor evoked potentials (MEPs) and the onset of electromyography (EMG). **(A)** The size of ES MEPs correlates with EMG onset of the erector spinae (ES) muscle in older participants. However, **(B)** the same correlation is not present in young participants. The abscissa shows the size of ES MEP at 40 ms prior to the actual recognition reaction time (RRT). ES MEPs are calculated as a % of the ES MEP obtained at baseline when participants were not expecting to react to a visual cue. The ordinate shows the EMG onset of the ES muscle with respect to the visual cue. Note that the size of ES MEPs is smaller in old participants showing greater delayed anticipatory postural adjustments (APAs).

To characterize the relationship of the timing of increased corticospinal excitability with the onset timing of APAs and ES activity for postural control, we correlated amplitudes of ES MEPs at −40 ms (the time point when the corticospinal excitability started to increase) and the difference in EMG onset between the ES and AD muscles (APA) as well as the EMG onset of the ES muscle during the rapid shoulder flexion task. We found that ES MEP at −40 ms correlated with the onset of ES EMG in the elderly cohort (rho = −0.52, *p* = 0.02; Figure 4A), but not in the young cohort (rho = 0.22; *p* = 0.34; Figure 4B). The results indicate that the ES muscle was activated later in the older participants with smaller MEP amplitudes prior to the rapid shoulder flexion task. ES MEP at −40 ms did not correlate with the onset of ES EMG with respect to AD EMG in either young (rho = 0.41; *p* = 0.07) or older participants (rho = −0.23; *p* = 0.32).

### Demographic Characteristics From Questionnaires of the Study Cohorts

Results of the IPAQ questionnaire demonstrated that the younger cohort on average per week had a higher MET output (5,773.6 ± 4,979.7 METs) than the elderly cohort (5,105.2 ± 5,262.7 METs; *Z* = −2.1, *p* = 0.04). There were more young adults categorized as having a high level of activity than older adults ($\chi_{\left(2\right)}^{2}$ = 10.52, *p* = 0.005). We found that the elderly cohort had a higher percentage of individuals with a fear of falling in the FES-I questionnaire (42.3%) compared with the young group who had no fear of falling (0%; χ^2^ = 13.02, *p* < 0.001). Older adults had more concerns of pain and discomfort compared with young adults (χ^2^ = 9.64, *p* = 0.008). There was no difference in EQ-VAS values between elderly (87.44 ± 11.1 out of 100, being the best health the person could imagine) and young groups (85.54 ± 9.44; *t*_(47)_ = 0.64; *p* = 0.523), suggesting that they subjectively viewed their health to be at the same level, see Table 1 for demographic results.

## Discussion

Our results demonstrate that corticospinal excitability of the ES muscle increases in response to a self-initiated perturbation 40 ms prior to the onset of the rapid shoulder flexion in a seated position in both young and elderly healthy participants. The amount of facilitation of the ES MEPs prior to the shoulder movement was the same between the young and the elderly groups. Older participants showed delayed APAs during the fast shoulder flexion task compared with the young participants as expected. Interestingly, ES MEPs at 40 ms prior to the shoulder flexion correlated with EMG onset of the ES muscle in the older participants; the same correlation was not present in the young participants. This suggests that the timing of increasing corticospinal excitability prior to a self-initiated perturbation becomes more relevant with ageing in modulating APAs of the trunk for maintaining postural stability. This is the first study, to the best of our knowledge, demonstrating corticospinal excitability modulating APAs of the trunk in the elderly and its relationship with the function of postural control.

### Corticospinal Excitability and APAs in the Elderly

Several lines of evidence have shown increased corticospinal excitability of a prime mover from 50–120 ms prior to voluntary limb movements in young adults (Chen et al., 1998; Leocani et al., 2000; Mackinnon and Rothwell, 2000; Kennefick et al., 2014). Trunk muscles are non-prime movers in a voluntary movement of the limbs and activated prior to or concurrent with movements of the limbs to maintain postural stability (Hodges and Richardson, 1997). Corticospinal excitability of the trunk muscles, specifically the ES muscles, have been shown to increase ~40 ms prior to the fast voluntary arm movements when young healthy adults were in standing and lying positions (Chiou et al., 2018). The results presented here are in line with these findings as the young participants showed increased corticospinal excitability at 40 ms prior to the shoulder flexion task in the seated position. Our findings further support the notion that increased corticospinal excitability of the ES in the APA window is hardwired to limb movement and independent from postures.

We found that APAs of the trunk were delayed in older participants during a rapid shoulder flexion task. This is in keeping with previous findings showing delayed APAs during self-paced voluntary movements in older adults (Woollacott and Manchester, 1993). What are the possible mechanisms underlying delayed APAs seen with ageing? Work has shown increased presynaptic inhibition in spinal circuits prior to gait initiation (i.e., during APAs) in older adults compared with young adults and levels of the inhibition correlating with amplitudes of APAs (Filho et al., 2021). Other studies also reported age differences in the modulation of presynaptic inhibition during an upper-limb postural task (Baudry et al., 2010) and standing (Degani et al., 2020). This suggests a relationship of delayed APAs with increased spinal inhibition. In addition, ageing has profound effects on the structure and function of the brain. Imaging studies reported reduced white matter integrity in older adults compared with young adults and this reduction in the brain structure associated with age-related changes in motor control (Coxon et al., 2012; Holtrop et al., 2014). Indeed, it is reported that increased volume of white matter hyperintensities correlates with the delayed reaction time of step initiation (Sparto et al., 2008). Since the cerebral cortex mediates APAs, age-related decline in the structure of white matter pathways may influence the timing of APAs. While our findings confirm delayed APAs in old adults, we did not observe a delay in the timing of increasing corticospinal excitability of the ES. Conversely, we found a similar corticospinal excitability profile in the APA window in both the elderly and young cohorts. Previous research using a simple reaction time task in a prime-mover thumb muscle showed that the timing of increasing corticospinal excitability was similar between young and elderly participants (Levin et al., 2011). However, they found higher corticospinal excitability in the elderly participants as compared to the young participants (Levin et al., 2011). As such, they concluded that this increased corticospinal excitability was a compensatory strategy used by the old adults to maintain motor performance. Evidence from a functional MRI (fMRI) study has shown a compensatory strategy of hyper-activation with increasing age, where additional cortical and subcortical areas increased in activation in older adults when compared with young whilst taking part in a simple motor task (Mattay et al., 2002).

In this study, we observed a similar corticospinal excitability profile in both timing and magnitudes between the older and young participants, though older participants reacted slower and had delayed APAs compared with the young participants. Our results may indicate that our elderly cohort did not use a compensatory strategy that involves increased corticospinal drive to the trunk muscles to maintain APAs in an RRT paradigm, which is more complex than the simple reaction time paradigm.

Interestingly, we found an age-specific correlation between the size of ES MEP at 40 ms prior to the shoulder movement and the onset of ES activation in the elderly participants. Since the activity of the ES muscle during the fast shoulder flexion task is to maintain postural stability, this result may imply that the timing for the motor cortical excitability reaching functional levels is relevant in modulating the onset timing of the ES muscle in response to a self-initiated perturbation in old age. Several lines of evidence suggest motor cortical modulation of postural control differ between young and old adults (Papegaaij et al., 2014, 2016; Watanabe et al., 2018a,b). Studies show reduced motor cortical inhibition in old individuals during standing on foam, compared to a rigid surface, and during unsupported, compared to supported forward lean posture (Papegaaij et al., 2014). Furthermore, prior work using coherence analysis of muscle activity in lower limbs showed a larger muscular coherence in the beta band in the elderly than in the young adults during one-leg stance (Watanabe et al., 2018b), meaning that there is a greater corticospinal drive to the contracting muscles of the standing leg in older adults compared with young adults. Our findings, together with these previous findings, highlight that the age differences in postural control are likely caused, at least in part, by changes in motor cortical modulation of such movements.

Our elderly cohort showed similar corticospinal drive to the ES muscle as to the young cohort but delays in onset timing of ES activity and APAs. An intriguing question is why the older participants who did not have a history of falls or were not clinically diagnosed as at high risk of falling were unable to increase the corticospinal drive to the ES muscle to maintain the function of APAs? An explanation is that delayed APAs with ageing cannot be improved by increasing corticospinal excitability. Previous studies reported reduced postural control in the elderly even though motor cortical activity was increased (Papegaaij et al., 2014, 2016). This suggests that the relationship between cortical modulation and postural control in old individuals may not solely depend on the corticospinal function. Evidence suggests subcortical neural circuits, such as reticulospinal pathways, also contributing to postural adjustments (Prentice and Drew, 2001; Schepens and Drew, 2004; Takakusaki, 2017). Research has shown the reticulospinal inputs being upgraded after spinal cord injury and in ageing as a compensatory mechanism to maintain motor control (Sangari and Perez, 2020; Maitland and Baker, 2021). Although the role of the reticulospinal circuits in modulating corticospinal excitability profile during the APA window remains unknown, it is possible that the reticulospinal circuits influence the control of APAs. It could be that older adults with little or no delay in APAs receive more inputs to the trunk muscles from the reticulospinal pathways. Furthermore, it is a possibility that spinal circuits contribute to the correlation between the ES MEP and the EMG onset of the ES muscle. Work has shown age-related differences in spinal excitability of a leg muscle during a unilateral arm raise task in standing (Hortobagyi et al., 2018). Using the same experimental paradigm we previously showed changes in intracortical inhibition corresponding to the changes in ES MEPs but no difference in the spinal excitability at any time points during APAs in young adults (Chiou et al., 2018). However, since we did not directly measure spinal excitability from the elderly cohort, we cannot rule out this possibility.

### Clinical Relevance

Previous research has shown the impairment of APAs and postural control in various populations, including the elderly (Woollacott and Manchester, 1993; Kanekar and Aruin, 2014), stroke patients (Pereira et al., 2014), Parkinson’s disease (Bleuse et al., 2008), and those with lower back pain (Mok et al., 2007; Masse-Alarie et al., 2012). An increased number of falls have been associated with a loss of postural control (Rogers et al., 1992), suggesting the vital role of postural control in the elderly population. Therefore, our findings have implications for the evaluation of postural control and for designing methods for treating APAs. Our results suggest the timing of increasing corticospinal excitability prior to a perturbation becomes more relevant with ageing in maintaining postural stability, highlighting the possibility of targeting the corticospinal function to improve postural control in old age. Furthermore, previous studies found improvements in APAs of leg and trunk muscles prior to both self-initiated and external perturbations following ball throwing exercise (Aruin et al., 2015, 2017). Differing from conventional balance and strengthening exercises, the ball throwing exercise requires motor planning and adjustments to external factors (i.e., perturbations). The M1 and other motor areas (e.g., supplementary motor area, premotor cortex) are involved in motor planning and execution (Dum and Strick, 2002). Given modulation of APAs being complex and likely receiving inputs from other neural circuits in addition to the corticospinal pathways, tasks involving multiple neural circuits might be beneficial for treating APAs and postural control. Further investigations are needed in translating the improvement of APAs into balance control in older people.

### Limitations and Future Recommendations

A limitation of this study was no direct measurement of subcortical or spinal circuits during the APA window. Animal studies have shown that reticulospinal pathways contribute to postural control and have projections to the corticospinal tract (Prentice and Drew, 2001; Schepens and Drew, 2004). Future research will clarify the influence of subcortical and spinal mechanisms in age-related changes in postural control. Moreover, it has to be noted that due to the nature of the dynamic task and a result of reorganizing the data based on the actual timing of the TMS pulse, the number of MEPs averaged for each time point during the APA window is less than 10 MEPs. This may increase the variation of the data. Additionally, since there were low levels of activity in the ES muscles prior to the shoulder movement to maintain the upright posture of the body, we cannot completely rule out the influence of ES activity on the amplitudes of MEPs in the ES muscle, albeit there was no significant difference in ES EMG across all time points prior to the shoulder movement.

## Conclusion

We demonstrate delayed APAs during a rapid shoulder flexion movement in the elderly cohort, despite a similar corticospinal excitability profile of the ES muscle in both elderly and young groups. However, corticospinal excitability at 40 ms prior to the shoulder movement correlated with the onset of ES activity counterbalancing the perturbations induced by the shoulder movement in older participants. Our findings provide evidence for corticospinal contribution to postural control in ageing and highlight the importance of targeting corticospinal function for improved postural control in the elderly.

## Data Availability Statement

The datasets presented in this article are not readily available because participant’s data need to be handled in accordance with the current data protection laws and ethical guidelines. Requests to access the datasets should be directed to s.chiou@bham.ac.uk.

## Ethics Statement

The studies involving human participants were reviewed and approved by University of Birmingham Ethics Committee (ERN_17-1541AP4). The patients/participants provided their written informed consent to participate in this study.

## Author Contributions

Study concept and design: all authors. Data acquisition and analysis: RR and S-YC. Drafting the manuscript and figures: RR and S-YC. Editing the manuscript and figures: NJ. Read and approved the final version of this manuscript: all authors. All authors contributed to the article and approved the submitted version.

## Conflict of Interest

The authors declare that the research was conducted in the absence of any commercial or financial relationships that could be construed as a potential conflict of interest.

## Publisher’s Note

All claims expressed in this article are solely those of the authors and do not necessarily represent those of their affiliated organizations, or those of the publisher, the editors and the reviewers. Any product that may be evaluated in this article, or claim that may be made by its manufacturer, is not guaranteed or endorsed by the publisher.
